# Can Parietin Transfer Energy Radiatively to Photosynthetic Pigments?

**DOI:** 10.3390/molecules23071741

**Published:** 2018-07-17

**Authors:** Beatriz Fernández-Marín, Unai Artetxe, José María Becerril, Javier Martínez-Abaigar, Encarnación Núñez-Olivera, José Ignacio García-Plazaola

**Affiliations:** 1 Department Plant Biology and Ecology, University of the Basque Country (UPV/EHU), 48940 Leioa, Spain; beatriz.fernandezm@ehu.eus (B.F.-M.); unai.artetxe@ehu.eus (U.A.); josemaria.becerril@ehu.eus (J.M.B.); 2 Faculty of Science and Technology, University of La Rioja (UR), 26006 Logroño (La Rioja), Spain; javier.martinez@unirioja.es (J.M.-A.); encarnacion.nunez@unirioja.es (E.N.-O.)

**Keywords:** anthraquinones, chlorophyll, fluorescence, parietin, photosynthesis, ultraviolet radiation, UV-B, *Xanthoria parietina*

## Abstract

The main role of lichen anthraquinones is in protection against biotic and abiotic stresses, such as UV radiation. These compounds are frequently deposited as crystals outside the fungal hyphae and most of them emit visible fluorescence when excited by UV. We wondered whether the conversion of UV into visible fluorescence might be photosynthetically used by the photobiont, thereby converting UV into useful energy. To address this question, thalli of *Xanthoria parietina* were used as a model system. In this species the anthraquinone parietin accumulates in the outer upper cortex, conferring the species its characteristic yellow-orange colouration. In ethanol, parietin absorbed strongly in the blue and UV-B and emitted fluorescence in the range 480–540 nm, which partially matches with the absorption spectra of photosynthetic pigments. In intact thalli, it was determined by confocal microscopy that fluorescence emission spectra shifted 90 nm towards longer wavelengths. Then, to study energy transfer from parietin, we compared the response to UV of untreated and parietin-free thalli (removed with acetone). A chlorophyll fluorescence kinetic assessment provided evidence of UV-induced electron transport, though independently of the presence of parietin. Thus, a role for anthraquinones in energy harvesting is not supported for *X. parietina* under presented experimental conditions.

## 1. Introduction

Anthraquinones are a wide family of lichen secondary metabolites, particularly conspicuous in the family Teloschistaceae, where they are frequently deposited as crystals outside the fungal hyphae, conferring species a characteristic yellow or orange colouration. While most secondary metabolites known from lichens (approximately 800) are exclusively synthesised by them, a couple of lichenic substances are found also in non-lichenised fungi or higher plants. Parietin is one of them, found in *Aspergillus* and *Penicillium* fungi and in *Ventilago*, *Rheum*, and *Rumex* among vascular plants [1]. The function of these compounds has been frequently studied using the deposition of parietin in *Xanthoria parietina* as a model system. From these studies a photoprotective role has been concluded [2], although protection from herbivory by snails [3] and anti-bacterial and -fungal activity [4] have also been demonstrated. The photoprotective hypothesis is consistent with the pattern of seasonal changes in parietin concentration, which peaks during the periods of higher irradiance [5]. While the protection against blue light seems unequivocal, the protective role against UV-B is not clear [6], since UV-B has little effect on lichen growth when parietin is removed [7]. This observation is consistent with the higher importance of wavelengths longer than UV-B in photoinhibition [8], but counterintuitive with the demonstrated requirement of UV-B to trigger parietin synthesis [7,9]. Whatever the exact photoprotective function of parietin is, this mechanism exemplifies the intimate action of both symbionts to avoid photoinhibition, in which the photobiont activates chloroplastic mechanisms of photoprotection [10] while the mycobiont synthesises parietin under the regulation of carbon acquired by the photobiont [11]. In fact, the mechanism is so efficient that it seems to be the basis of the extraordinary stress tolerance of lichens. For example, *Xanthoria elegans* is able to recover after exposure to the extreme conditions of outer space (massive UV radiation, cosmic rays) [12]. Furthermore, the protection exerted by anthraquinones is associated with the evolutionary success and diversification of the family Teloschistaceae [13].

Apart from the capacity of these compounds to scatter or absorb harmful blue and UV radiation excesses, most of them present the property of emitting visible fluorescence. The emission of fluorescence within the visible range is an uncommon property in nature, with chlorophyll among the most remarkable fluorescent molecules in photosynthetic organisms. However, in the case of lichen metabolites, fluorescence is not that unusual and many biochemically unrelated compounds are fluorescent [14]. The overlapping between the emission spectra of fluorophores and the action spectrum of chlorophyll led Rao and LeBlanc [15] to propose a possible role in the conversion of damaging radiation into harmless light that can be photosynthetically used by the photobiont. A similar process of radiative energy transfer has been demonstrated in angiosperms [16] such as spruce (*Picea abies*) [17] and winter triticale (x *Triticosecale*) [18], where it is linked to the presence of fluorescent ferulic acid in the epidermis [19,20]. This mechanism could explain why so many lichen secondary compounds are fluorescent, but a demonstration of this physiological effect in lichens remains unknown. Thus, in the present work we wondered whether lichen secondary metabolites may transfer radiative energy to photosynthetic pigments and if fluorescence itself could represent an adaptive trait that conferred any biological advantage. To address this question, field-collected thalli of *X. parietina* were used as a model system.

## 2. Results and Discussion

**Figure 1:**
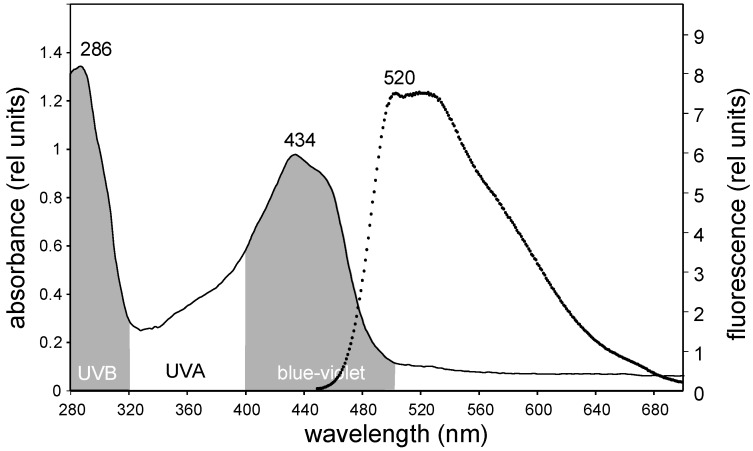
Overlapped absorbance (continuous line) and fluorescence spectra (discontinuous line) of acetone extracts from sun-collected thalli of *X. parietina,* resuspended in pure ethanol. Excitation wavelength for fluorescence emission was 430 nm, the wavelength at which the highest fluorescence yield was observed.

Absorption spectra of acetonic extracts from *X. parietina* resuspended in ethanol showed two maxima, one in the UV-B region (at 286 nm) and a second one in the blue region (at 434 nm, Figure 1). These two peaks are fully consistent with the proposed light shielding role of these compounds, which would be more prominent in the UV or blue range of the spectrum, corresponding with the more potentially damaging wavelengths [8]. These extracts would be likely dominated by parietin, but other anthraquinones can also be present [21]. Since the exact composition of the extract is not relevant for their protective function, for simplicity, and in agreement with previous literature, the term “parietin” will be used herein to denote the blend of acetone-extractable compounds. Ethanol-resuspended extracts were fluorescent (Figure 1), with a peak of maximum emission in the green-yellow region (520–540 nm) after excitation at 430 nm.

**Figure 2:**
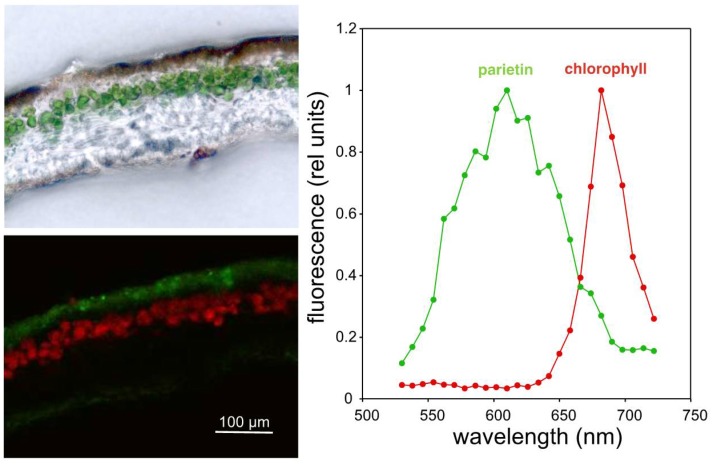
(**Left**): Bright field (**top**) and confocal (**bottom**) images of a cross section of *X. parietina*. Arbitrary colours (red and green) identify distinct emission spectra from chlorophyll and parietin, respectively. (**Right**): Fluorescence emission spectra of parietin and chlorophyll.

Confocal laser microscopy confirmed these optical properties in vivo (Figure 2), although for parietin there was a shift towards the orange region, with a maximum emission at 610 nm. This observation also revealed that fluorescent parietin accumulates only in the outer cortex of the lichen thalli (Figure 2), with little or no contact with the algal layer underneath. This red-shift in fluorescence with respect to the acetone extracts could suggest that parietin is bound to proteins, as occurs in chlorophylls that are strongly red-shifted in the fluorescence emission in vivo [22]. However, this is unlikely the case of parietin since this compound forms extracellular crystals, and no protein has been identified to bind specifically to parietin. Despite the fact that this fluorescence emission does not correspond with the maximum in the action spectrum of photosynthesis in green alga, the photobionts of *X. parietina*, photons emitted towards the algal layer, could, in principle, be used photosynthetically with relatively high efficiency [23]. If this process of radiative energy transfer occurs, it would extend the range of photosynthetically active wavelengths to the UV-A and UV-B ranges. Interestingly, parietin is only one example among the wide diversity of fluorescent lichen metabolites. In fact, the diverse biosynthetic routes of lichen secondary metabolites converge into the generation of yellow-coloured fluorescent pigments that can be found in several evolutionary lineages of lichens. This is, for example, the case of rhizocarpic acid, a shikimic acid derivative found in the order Lecanorales [24], with a similar location within the lichen thallus and comparable optical properties (Figure S1). Interestingly, both parietin and rhizocarpic acid are protective enough as to assure survival in the conditions of outer space [12].

**Figure 3:**
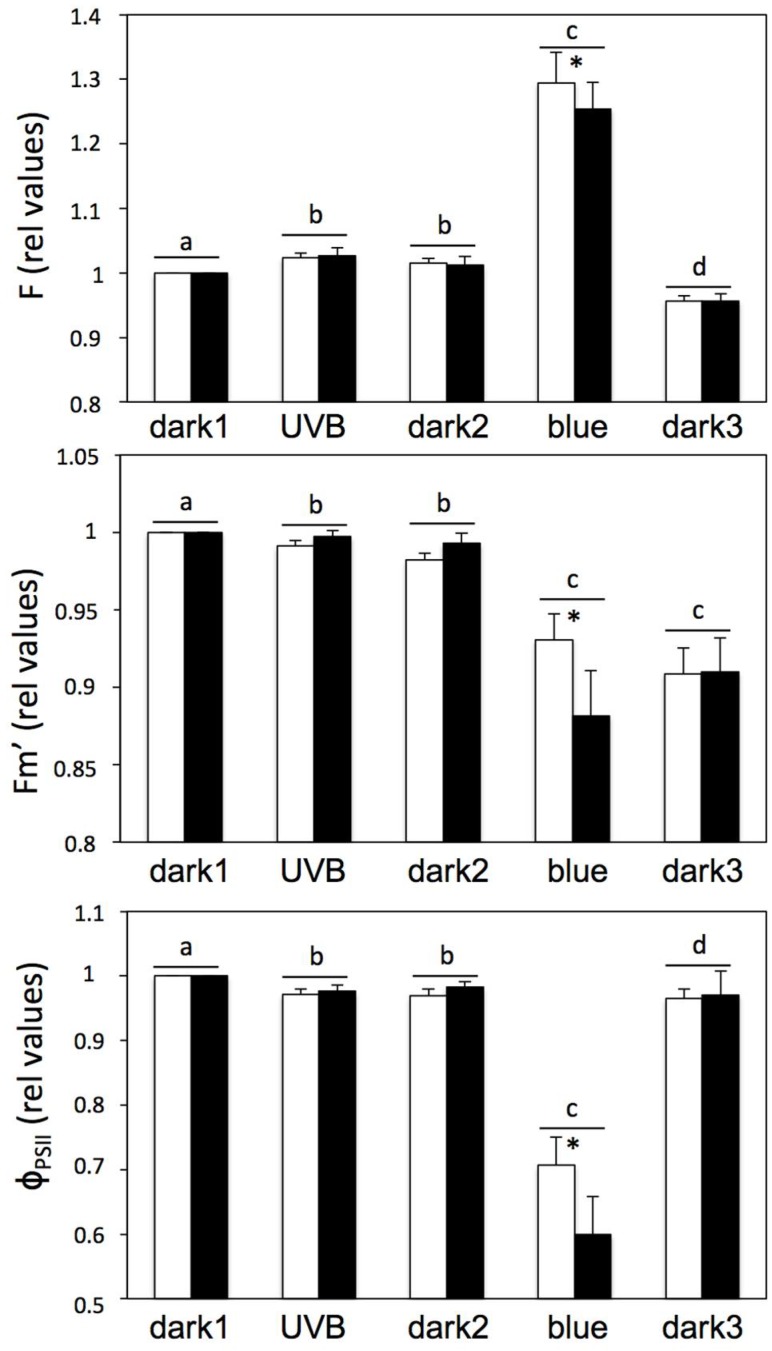
Effect of UV-B and blue light treatments on F, Fm’, and φ_PSII_ on thalli with (open bars) or without (closed bars) parietin, following the kinetic assay described in Supplementary Figure S2. Control values (first measurement in darkness) for φ_PSII_ were 0.588 ± 0.020 and 0.532 ± 0.044 (nonsignificant differences) for samples with and without parietin, respectively. Data are average ± SE (*n* = 6). Significant differences among light treatments are indicated with different letters and between thalli with or without parietin with an asterisk (*p* < 0.05).

To test the existence of photon transfer from parietin to chlorophyll, the photochemical response to realistic UV-B and blue light irradiances of untreated thalli, estimated by chlorophyll fluorescence kinetics, was compared with that of parietin-free thalli (pretreated with acetone for its removal) following dark and subsequent exposure to UV-B and blue light (Figure S2). Exposure to UV-B triggered a slight, but significant, decrease in φ_PSII_ and a slight but significant enhancement of the steady-state fluorescence (F) (Figure 3), indicating a certain activation of photochemical processes. Since UV-B LEDs did not emit in the visible range (Figure S3), this photochemical process was caused exclusively by the action of UV-B. However, these patterns were observed irrespective of the presence of parietin, and its relative magnitude was even slightly (but not significantly) higher when parietin was removed (Figure 3). Blue light also induced the same pattern of changes (to a higher extent), implying the activation of a process of non-photochemical quenching of chlorophyll fluorescence. This pattern was again more noticeable in thalli without parietin (Figure 3) and these changes were not fully reversible when thalli returned to darkness. The immediate enhancement of F when thalli are exposed to UV-B (Figure S2) indicates that the obtained observations cannot be explained by a process of UV-induced damage, which would generate a progressive increase on this parameter. Taken together, all these observations clearly demonstrate that parietin contributes to the attenuation of blue and UV-B radiation, but fail to support any notion of energy transfer between parietin and chlorophyll.

Overall, these observations suggest that UV-B is able to activate photochemical activity on PSII, but this activity is not mediated by the presence of parietin. The presence of fluorescent compounds of unknown nature in the thallus [25] could be the basis for such UV-B-induced activation of photochemical processes, whose contribution to the overall CO_2_ gain remains to be established. Furthermore, parietin itself seems to attenuate these responses in agreement with its well-known UV-B protective role.

## 3. Materials and Methods

### 3.1. Lichen Collection

Specimens of *X. parietina* growing on the south-facing bark of deciduous trees (high light exposure) were collected in the surroundings of the University Campus of Leioa (Lat 43°20′ N; Long 2°58′ W). Thalli of *A. hilaris* were collected from quartzite rock from La Rioja (Lat 42°15′ N; Long 2°45′ W). Parietin was removed from living lichens using the method described by [26], that is, based on the easy extraction of parietin from dry lichens with acetone. An undesirable side-effect of acetone treatment on membrane integrity has been recently described for *X. parietina* and other species [27]. However, despite the finding of a significant leakage from the membrane, no deleterious effect was observed on chlorophyll fluorescence in *X. parietina*. In the present study (Figure 3), Fv/Fm showed a nonsignificant decrease after parietin removal. This was probably caused by the damaging effects of acetone [27]. However, for the main purpose of the paper—testing the hypothesis of energy transfer between parietin and photosystems—this effect was not relevant, since photochemical efficiency was maintained at 90%.

### 3.2. Chlorophyll Fluorescence

Chlorophyll *a* fluorescence was measured using a portable modulated fluorometer PAM 2500 (Walz, Effeltrich, Germany). The maximum Chl fluorescence yield (Fm or Fm’) was induced with a saturating pulse (7795 μmol photons m^−2^ s^−1^) while minimum fluorescence (Fo) was recorded with low measuring light intensities and F represented the steady-state fluorescence under any light condition. The actual yield (φ_PSII_ or ΔF/Fm’) was (Fm’ − F)/Fm’. The kinetic protocol described in Figure S2 (120 s darkness, 60 s UV-B, 120 s darkness, 70 s blue light, 100 s darkness) was applied to thalli containing or not containing parietin (removed by acetone washing).

### 3.3. Confocal Microscopy

Naturally present secondary lichen compounds can be used as fluorophores in confocal microscopy to characterise the structure of the lichen thallus and the location of these compounds [28]. Fresh samples were frozen in OCT medium (Tissue-Tek^®^ OCT Compound, Sakura Finetek Europe B.V., Alphen aan den Rijn, The Netherlands) and 15 μm sections were obtained in a Cryostat (Thermo Shandon FSE, Waltham, MA, USA). XY lambda scans of fresh sections were performed in a Leica LCS sp2 AOBS confocal microscope (Leica Instruments, Exton, PA, USA) using an HCX PL APO CS 40× immersion oil lens (NA = 1.25). Excitation was set at 458 nm and lambda scans ranged from 530 to 730 nm. Emission spectra from the regions of interest were obtained using ImageJ [29].

### 3.4. Fluorescence and Absorbance Spectra

Parietin was gently extracted from dry individuals of *A. hilaris* and *X. parietina* with pure acetone as described in [26]. Absence of chlorophyll in these extractions was verified by measuring absorbance in the red region (650–680 nm). Later, acetone was evaporated and the precipitate resuspended in pure ethanol. Absorbance spectra were obtained with a Multiskan Spectrum spectrophotometer (Thermo Electron Corporation, Vantaa, Finland) in the wavelength range of 280–700 nm. Fluorescence emission and excitation spectra were performed over the same extracts with a Bowman Series2 luminescence spectrometer (SLM AMINCO, SLM Instruments, Inc., Rochester, NY, USA). Spectral irradiance was measured with a spectroradiometer (Macam SR9910, Macam Photometrics Ltd., Livingstone, UK).

### 3.5. UV-B and Blue Light Experiments

To study energy transfer from parietin, the response of untreated thalli to UV-B was compared with that of parietin-free thalli (pretreated with acetone for its removal). Thalli were exposed to UV-B or blue light, and the corresponding absorption maxima of parietin and chlorophyll fluorescence were monitored to estimate electron transport (an example of fluorescence kinetics is shown in Supplementary Figure S2). Illumination was provided by LEDs (peak emission at 300 and 450 nm for UV-B and blue light, respectively). UV-B LEDs did not emit photons in the visible range. The UV-B irradiance received by the lichen surface was 1.13 W m^−2^. This value was chosen to correspond to the UV-B irradiance at solar noon in spring (April–May) at a typical mid-latitude and low-altitude place [30] (Supplementary Figure S3).

### 3.6. Statistical Analyses

Data showed heteroscedasticity. Significant differences in chlorophyll fluorescence parameters among light treatments were checked with a nonparametric Kolmogorov–Smirnov test. Significant differences between thalli with or without parietin, within each light treatment, were checked with a Wilcoxon paired test. All statistical analyses were conducted at alpha < 0.05 with the SPSS v20 package (IBM Corp., Armonk, NY, USA).

## Supplementary Materials

The following are available online at http://www.mdpi.com/1420-3049/23/7/1741/s1, Figure S1: Overlapped absorbance and fluorescence spectra of acetone extracts from sun-collected thalli of *Acarospora hilaris* resuspended in pure ethanol. Figure S2: Effect of parietin removal and an example of a fluorescence kinetic assay. Figure S3: UV-B LED emission spectrum.
