# Supplementary material for: Can Parietin Transfer Energy Radiatively to Photosynthetic Pigments?
Source: Molecules. 2018 Jul 17;23(7):1741. doi: 10.3390/molecules23071741 (PMC6099737; doi:10.3390/molecules23071741)
Supplement: Supplementary file 1 [file molecules-23-01741-s001.pdf]

Supplementary Materials

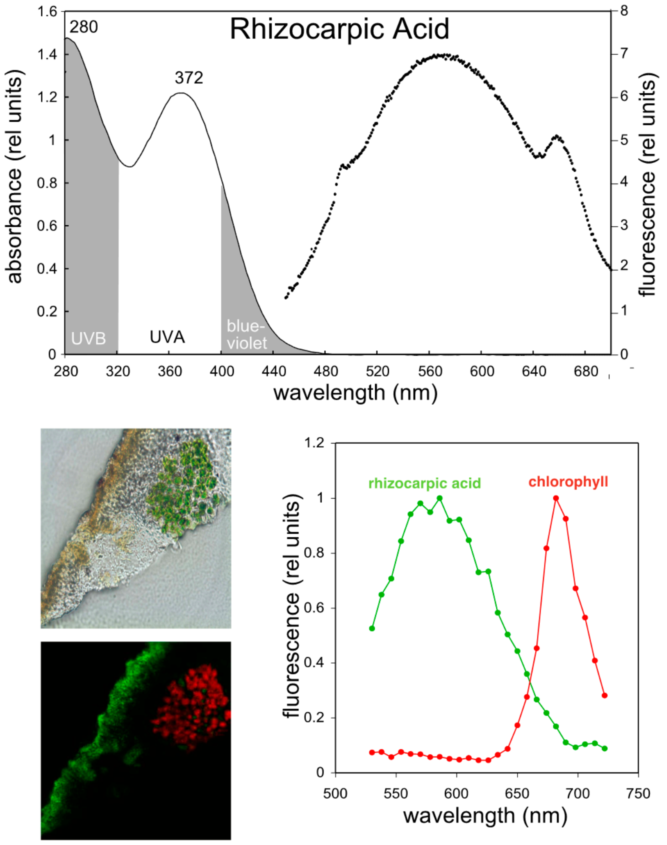

Figure S1. Top: Overlapped absorbance and fluorescence spectra of acetone extracts from sun-collected thalli of *Acarospora hilaris* resuspended in pure ethanol. Excitation wavelength for fluorescence emission was 435 nm, the wavelength where the highest fluorescence yield was observed. Bottom left: Bright field and confocal images of a cross section of *A. hilaris*. Arbitrary colours (red and green) identify distinct emission spectra from chlorophyll and rhizocarpic acid, respectively. Bottom right: fluorescence emission spectra of chlorophyll and parietin.

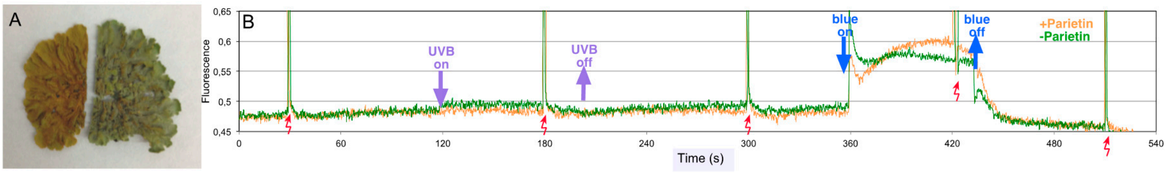

Figure S2. A. Effect of parietin removal from half a thallus. B. Example of a typical fluorescence kinetic assay. Thalli (with and without parietin) were kept in darkness prior to one minute of UVB illumination. After recovery in darkness, thalli were exposed blue light (200 µmol photons m^{-2} s^{-1}). Saturating pulses (red arrows) were applied at the end of each illumination interval.

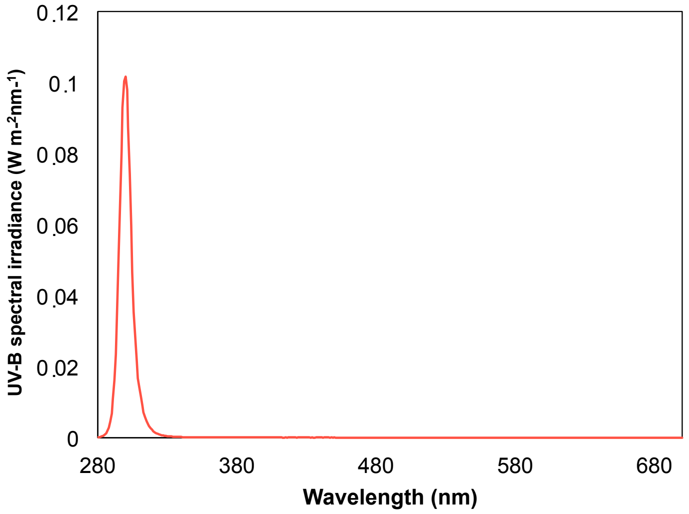

Figure S3. UVB-LED emission spectrum measured at the same distance as the samples.
